# Neural Correlates of Direct and Indirect Suppression of Autobiographical Memories

**DOI:** 10.3389/fpsyg.2016.00379

**Published:** 2016-03-18

**Authors:** Saima Noreen, Akira R. O’Connor, Malcolm D. MacLeod

**Affiliations:** ^1^Department of Psychology, Goldsmiths, University of LondonLondon, UK; ^2^School of Psychology and Neuroscience, University of St AndrewsSt Andrews, UK; ^3^School of Natural Sciences, University of StirlingStirling, UK

**Keywords:** think/no-think, memory retrieval, direct suppression, fMRI, autobiographical memories

## Abstract

Research indicates that there are two possible mechanisms by which particular target memories can be intentionally forgotten. Direct suppression, which involves the suppression of the unwanted memory directly, and is dependent on a fronto-hippocampal modulatory process, and, memory substitution, which includes directing one’s attention to an alternative memory in order to prevent the unwanted memory from coming to mind, and involves engaging the caudal prefrontal cortex (cPFC) and the mid-ventrolateral prefrontal cortex (VLPFC) regions. Research to date, however, has investigated the neural basis of memory suppression of relatively simple information. The aim of the current study was to use fMRI to identify the neural mechanisms associated with the suppression of autobiographical memories. In the present study, 22 participants generated memories in response to a series of cue words. In a second session, participants learnt these cue-memory pairings, and were subsequently presented with a cue word and asked either to recall (think) or to suppress (no-think) the associated memory, or to think of an alternative memory in order to suppress the original memory (memory-substitution). Our findings demonstrated successful forgetting effects in the no-think and memory substitution conditions. Although we found no activation in the dorsolateral prefrontal cortex, there was reduced hippocampal activation during direct suppression. In the memory substitution condition, however, we failed to find increased activation in the cPFC and VLPFC regions. Our findings suggest that the suppression of autobiographical memories may rely on different neural mechanisms to those established for other types of material in memory.

## Introduction

Remembering past events is something we all engage in. While such recollections are for the most part enjoyable, there are some occasions when we might prefer not to bring them to mind. Traumatic or upsetting events in one’s lives often tend to be associated with negative feelings and emotions which we would prefer to avoid. Thus, the ability to expel upsetting memories from conscious awareness confers obvious advantages for the rememberer. Recent psychological research has sought to establish the extent to which we possess executive control over which memories come to mind, and the mechanisms responsible. Using a wide range of materials, it is now well-established that the repeated attempt to consciously forget unwanted memories impairs their subsequent recall, although debate continues about the nature of the underlying mechanism responsible for such forgetting (Anderson and Green, 2001; Anderson et al., 2004, 2011; Bergström et al., 2009; Hanslmayr et al., 2010; Noreen and MacLeod, 2013, 2014, 2015; Noreen et al., 2014).

Most recently, this systematic forgetting effect has been demonstrated during the retrieval of autobiographical memory – memory for events that have been personally experienced (Noreen and MacLeod, 2013, 2014; Stephens et al., 2013). Using the autobiographical think/no-think task (ATNT), Noreen and MacLeod (2013) asked participants to generate positive and negative memories in response to a series of neutral cue words. In a subsequent session, participants were asked to recall some of the memories (‘think’ condition), or to avoid saying or thinking about other memories (‘no-think’ condition). In a final test of memory, participants who had been repeatedly instructed to ‘not think’ about particular memories were found to have poorer recall for details associated with these memories than baseline memories that had not been the subject of either ‘think’ or ‘no-think’ instructions. Importantly, although participants still remembered that the events had happened to them, systematic forgetting effects emerged for details associated with those events they had been instructed to prevent coming to mind.

These kinds of forgetting effects have been largely interpreted within a suppression framework in which inhibitory control is thought to disrupt the availability of the representation of the unwanted memory, thereby rendering it inaccessible to subsequent retrieval (Anderson and Green, 2001; Anderson, 2003; Anderson and Hanslmayr, 2014). Imaging studies have allowed for greater speculation as to how this kind of inhibition comes about. Specifically, Anderson et al. (2004) found that the act of memory suppression engaged the dorsolateral prefrontal cortex [DLPFC; Brodmann area (BA) 46/9], plus a reduction in activation in the hippocampus – a region thought to be involved in conscious recollection (Eldridge et al., 2000; Eichenbaum et al., 2007). Critically, this reduction in hippocampal activity during attempts to suppress has not only been found when compared to the ‘think’ condition, but also in comparison to the baseline fixation condition (Depue et al., 2007; Levy and Anderson, 2012).

According to Anderson et al. (2004), hippocampal activity is modulated by executive control via the DLPFC with increased activation in this region being interpreted as an active executive process that inhibits unwanted memories from entering conscious awareness. In support of this notion, a negative relationship has been found between activation in the DLPFC and hippocampal activity during the ‘no-think’ condition (Depue et al., 2007, 2010; Benoit et al., 2014). Furthermore, effective connectivity analyses have shown a top–down modulatory influence of DLPFC on the hippocampus (Benoit and Anderson, 2012; Gagnepain et al., 2014), with negative coupling from the DLPFC predicting the magnitude of the forgetting effect (Benoit and Anderson, 2012; Benoit et al., 2014; Depue et al., 2016). Taken together, these findings provide converging evidence in support of a direct suppression mechanism which disengages retrieval processes supported by the hippocampus.

There is, however, a growing appreciation that forgetting effects for ‘no-think’ items are not always a function of inhibition. For instance, an unwanted memory could be excluded from conscious awareness by bringing an alternative memory to mind; that is, blocking rather than suppression *per se* (Hertel and Calcaterra, 2005). Indeed, some studies have shown that suppression effects can be strengthened by using a strategy to constrain attention (e.g., Hertel and Calcaterra, 2005; Hotta and Kawaguchi, 2009; Joormann et al., 2009). Utilizing the TNT paradigm, Hertel and Calcaterra (2005) demonstrated higher levels of forgetting when participants were provided with substitute words to think about instead of to-be-forgotten neutral words, thereby suggesting that memory substitution may be a useful strategy to help individuals to forget unwanted memories – although such substitution may, under certain circumstances, lead to hyperaccessibility of those memories one wishes to forget (Wegner and Zanakos, 1994).

Recently, the neural mechanisms underlying memory substitution have also come under scrutiny. Benoit and Anderson (2012), for instance, asked some participants to think of an alternative memory in order to help prevent an unwanted memory from coming to mind, whilst others were asked to directly ‘push’ the unwanted memory from conscious awareness. Their study found that, whilst both groups demonstrated comparable forgetting effects at final test, each condition involved distinct neural networks. Consistent with previous research, the direct suppression condition involved recruitment of the right DLPFC region which exerted a negative influence on hippocampal activation (Anderson et al., 2004). Memory substitution, on the other hand, was found to engage the caudal PFC (cPFC; approximating BA 44/9) and the mid-ventrolateral PFC (mid-VLPFC; approximating posterior BA 45). These two areas are thought to be involved in the retrieval of weaker memories when faced with interference from stronger memories (Wimber et al., 2008), and also during post-retrieval selection between active memory representations (Badre and Wagner, 2007; Kuhl et al., 2008).

Furthermore, effective connectivity analyses revealed that, in the direct suppression condition, the right DLPFC was negatively coupled with the hippocampus whereas, in the memory substitution condition, activation in the left cPFC and mid-VLPFC predicted greater hippocampal activation during no-think trials, suggesting that the hippocampus was recruited to keep the substitute memory in mind (Benoit and Anderson, 2012). This would suggest that direct suppression and memory substitution involve distinct neural networks that make different processing demands on the hippocampus.

The interaction between the frontal and hippocampal regions during suppression has been observed with a range of materials (Anderson et al., 2004; Depue et al., 2007; Butler and James, 2010; Levy and Anderson, 2012; Paz-Alonso et al., 2013; Gagnepain et al., 2014). No research to date, however, has examined whether this fronto-hippocampal interaction extends to the direct suppression of autobiographical memories. Furthermore, no research to date has explored the neural mechanisms underpinning memory substitution for autobiographical memories.

These are important issues to explore given the highly complex and personal nature of such memories. Autobiographical memory is considered to be a unique form of memory that enables the rememberer to make sense of the past. Indeed, such memories tend to comprise mental constructions of experienced events that are embedded in a rich context and are integrated with the rememberer’s perspective, evaluation and interpretation that serve to create a personal history (Conway, 2000).

Given the polylithic nature of autobiographical memories, the kind of forgetting associated with such memories may be inherently different from the kind of forgetting observed for other materials. Consistent with this view, a number of recent studies have found that, while participants systematically forget some of the details related to autobiographical events in ‘no-think’ condition, the events themselves were not forgotten following ‘no-think’ instructions (Noreen and MacLeod, 2013, 2014; Stephens et al., 2013). This suggests that different components within a memory may be differentially susceptible to the effects of suppression. This is in contrast to the kind of forgetting observed for relatively simple words or images whereby the entire representation of the unwanted memory trace appears to be inhibited (Anderson and Green, 2001; Anderson et al., 2004; Depue et al., 2007).

Additional support for the notion that the mechanism underlying autobiographical memory suppression may be different from the suppression of other kinds of memories comes from recent research by Hu et al. (2015). In their study, participants encoded memories by enacting a crime and received instructions to suppress the memory of the crime whilst EEG brain wave activity was recorded. Participants also completed an autobiographical implicit association test. Whilst suppression was found to reduce automatic cognitive biases associated with autobiographical memories, the neural signal in N200 was absent. Given that frontal N200 activity is an electrophysiological marker of cognitive control that has previously been found to be sensitive to suppression (Bergström et al., 2009; Mecklinger et al., 2009), the failure to find activity in the N200 region may indicate that the suppression of autobiographical memories is not reliant on the same pattern of fronto-hippocampal activation as the suppression of other kinds of memories. According to Hu et al. (2015), one reason for the failure in N200 activity may relate to the fact that participants engaged in suppression continuously throughout the memory task which may have made it more difficult to detect suppression in a trial-specific manner. Thus, it remains unclear as to whether the suppression of autobiographical memories relies on the activation of a different neural pathway or not.

In the present fMRI study, we set out to explore both direct suppression and memory substitution mechanisms underlying intentional forgetting using the ATNT task. Based on Noreen and MacLeod (2013, 2014) findings, we predicted that participants would show systematic forgetting effects for those details of autobiographical memories which they had previously been instructed to suppress (i.e., no-think condition). Additionally, we expected similar forgetting effects to emerge where participants had been encouraged to engage in thought substitution (Benoit and Anderson, 2012). Our rationale for including both a suppression and a thought substitution condition was to establish whether the two separate systems previously identified during the inhibition and substitution of relatively simple memories extended to the retrieval of complex autobiographical memories. We reasoned that, if memory suppression is not dependent upon the type of material being suppressed then we could expect: (i) the no-think condition to show increased activity in the right DLPFC region exerting a negative influence on hippocampal activation; and (ii) the memory substitution condition to show increased activation in the cPFC and mid-VLPFC regions. If, however, the suppression of autobiographical memories is unique to event-specific personal memories, then we could expect our findings to be consistent with those of Hu et al. (2015); that is, we could expect to find no frontal activation during direct suppression.

## Materials and Methods

### Participants

We initially had 34 participants (9 males; 25 females) attending the University of St Andrews, Scotland (aged 18–29 years) agree to take part in the study. We excluded 12 participants from the study as they failed to generate memories for all of the cue words, or had a history of, or were currently experiencing depression. This resulted in 22 right-handed participants (4 males; 18 females) taking part in both sessions of the study between 7 and 14 days apart (mean number of days = 10). The study was approved by the School of Psychology and Neuroscience Ethics Committee of the University of St Andrews and NHS Tayside Committee on Medical Research Ethics, Ninewells Hospital and Medical School. Upon obtaining informed consent, participants’ eligibility to participate in the study was confirmed using a short screening interview and a safety questionnaire routinely used by anyone undergoing an MRI scan at Ninewells Hospital, Dundee, UK. Participants were screened for current or previous history of any psychological or neurological disorders, and current levels of depressive symptomatology were measured using the Beck Depression Inventory-II (BDI-II, Beck et al., 1996).

### Procedure

#### Session One

**FIGURE 1:**
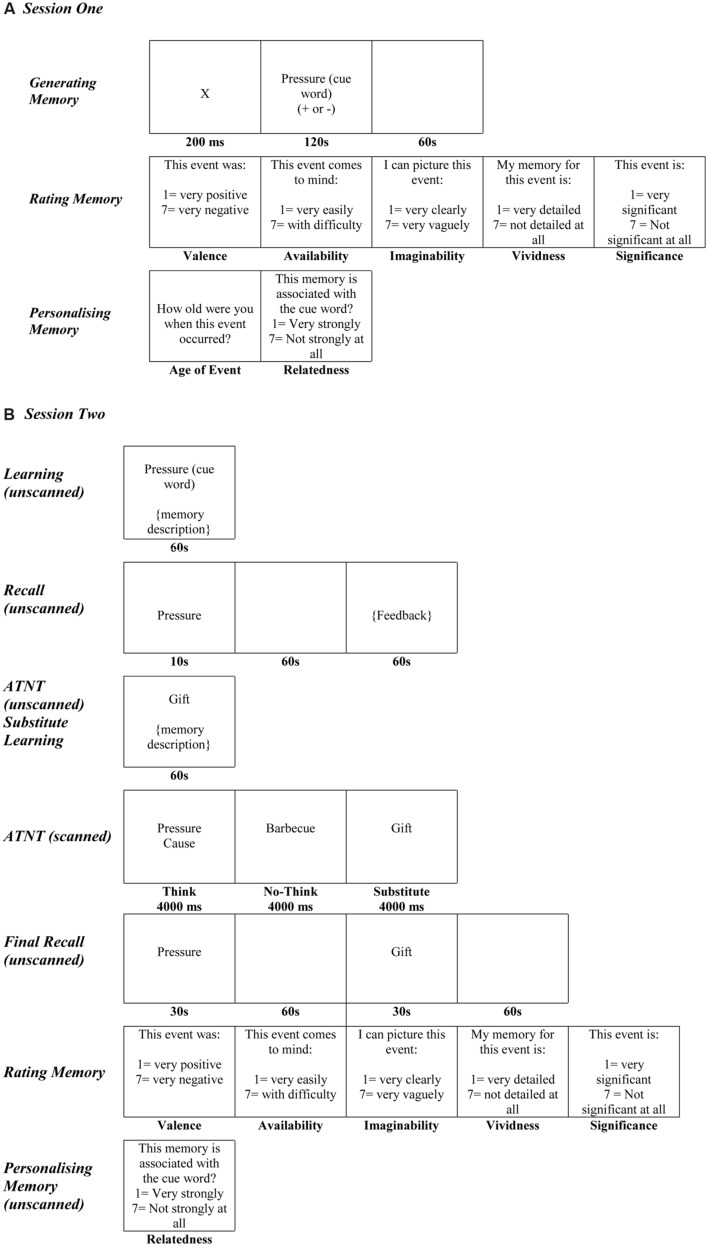
**(A)** Experimental procedure for session 1. **(B)** Experimental procedure for the ATNT task in session 2.

The first session took place in the laboratories at the School of Psychology and Neuroscience, University of St Andrews, and took approximately 2-3.5 h to complete. Participants were presented with a series of 34 neutral words [taken from Bradley and Lang, 1999; Affective Nouns for English Words (ANEW)] which were matched for valence, arousal, dominance, and frequency. Thirty words were used as cue words for the main study and the four additional cue words were used as practice trials in the second session. The 30 words used in the main study were divided into five sets of six items (three positive and three negative), with each set assigned to ‘think,’ ‘memory substitution,’ baseline,” and both ‘no-think’ conditions. The assignment of each word to each condition was duly counterbalanced across all participants. Participants were presented with a cue word which was accompanied by a plus (+) or minus sign (-). When presented with a plus sign, participants were instructed to think of a positive memory; and when presented with a minus sign, participants were instructed to think of a negative memory. Half the cue words were accompanied by a plus sign, and the other half were accompanied by a minus sign. Participants were given 2 min in which to generate a specific memory of an event from any period of their life in response to the cue word. Once participants had thought of a particular memory, they were given 1 min in which to describe the cause and the consequence(s) of the event. All memory descriptions were recorded. Participants were then asked to rate each memory regarding its perceived valence (1 = very positive and 7 = very negative), availability (1 = very easily and 7 = with difficulty), imaginability (1 = very clearly and 7 = very vaguely), vividness (1 = very detailed and 7 = not detailed at all), and significance (1 = very significant and 7 = very significant at all). Participants were also asked to estimate their age at the time the event occurred. Finally, participants were asked to rate how strongly they felt the memory they had generated was associated with the cue word (1 = very strongly and 7 = not strongly at all). See **Figure 1A**.

Once a memory had been recalled for each of the cue words, participants were told they would again see the same cue words, but that their task this time would be to generate a second set of memories. Participants were told that it was very important that they retrieved a new memory which was independent of, and unrelated to the first memory. For the generation of the second set of memories, the cue words that had previously been accompanied by a minus sign were accompanied by a plus sign to indicate that they should recall a positive memory. Similarly, cue words that had been previously accompanied by a plus sign were accompanied by a minus sign to indicate that they should recall a negative memory. Prior to Session Two, both sets of memories (68 in total) were transcribed and coded by the experimenter in relation to the perceived cause and consequence(s) of the event.

#### Session Two

The second session took place at the Clinical Research Centre, Ninewells Hospital, Dundee. The initial and final phases of the study did not involve scanning and were completed in a room with a computer in the Clinical Research Centre. The main think/no-think phase, however, involved the participant completing the task while being scanned. The second session took approximately 3–4 h to complete, with the main scanning session lasting approximately 28 min.

##### Learning phase (unscanned)

Participants were presented with a cue word for 60 s. As soon as the cue word appeared on the computer screen, participants were provided with a verbatim account of one of the memories they had previously generated, and were instructed to remember the details of that particular memory. Half of the participants were presented with the first set of memories generated in response to the cue words, and the other half were presented with the second set of generated memories. Regardless of the set of memories presented, all participants learnt 15 positive and 15 negative memories. A 500 ms inter-trial interval preceded the presentation of each cue word.

##### Recall phase (unscanned)

Once all of the cue words had been presented, participants were given a recall test. In this test, participants were presented with a cue word for 10 s and instructed to press the spacebar as soon as the associated memory from the learning phase came to mind. Participants were then given 60 s in which to recall the cause and the consequence(s) of the event. Feedback was provided on the accuracy of the memory recalled. Regardless of accuracy, participants were given 60 s to study the verbatim account of the memory. All participants achieved a minimum of 85% on this assessment.

##### Practice autobiographical think/no-think (ATNT) phase (unscanned)

Once participants had learnt the cue-memory pairings, they were told that they would see some of the cue words which would be presented in either a red or green font. Green cue words were accompanied by either the word ‘cause’ or ‘consequence’ and participants were told that their task was to think about the particular aspect of the memory (i.e., ‘think’ condition). For the red cue words, participants were told to not think about the associated memory (i.e., ‘no-think’ condition). In order to accomplish this, participants were told that they should block the memory from coming to mind, but not by replacing it with other memories or thoughts (Bergström et al., 2009). Two of the filler cue words appeared in green (one presented four times, and the other presented six times) and two filler cue words appeared in red (one presented four times, the other presented six times), thereby resulting in 20 practice trials in total.

##### Substitute learning phase (unscanned)

Immediately after the Practice ATNT phase, participants were presented with six of the cue words, which were accompanied by the second set of memories they had previously generated. Participants were presented with each cue word for 60 s and given a verbatim account of the new memory (i.e., the alternative memory generated in the first session for that cue word) and were told to try to remember all the details of that particular memory. They were told to learn the pairing in whatever way they preferred but to never think about the original memory.

##### Main autobiographical think/no-think phase (scanned)

Participants were then transferred to the fMRI scanner bed where, after undergoing a series of structural scans (see fMRI Acquisition and Preprocessing), they underwent the main ATNT phase of the study whilst being scanned. Participants were given a total of 288 experimental trials across four blocks which included ‘think’ and ‘no-think’ cue words in blocks 1 and 2 each being presented 12 times and ‘no-think’ and ‘memory substitution’ cue words in blocks 3 and 4 each being presented 12 times. In each block, the 72 experimental trials (each consisting of a 4000 ms presentation followed by an ISI of 500 ms) were intermixed with 18 passive fixation trials (4500 ms duration), yielding a total run time of approximately 7 min for each block.

The first two blocks consisted of 144 experimental trials (72 ‘think’ trials and 72 ‘no-think’ trials) in which ‘think’ and ‘no-think’ trials were intermixed. Participants were given 12 presentations of six cue words from each of the ‘think’ and ‘no-think’ conditions. For each trial, a cue appeared on the screen for 4000 ms in either green (‘think’) or red (‘no-think’) followed by an ISI of 500 ms. For the ‘think’ trials, cue words were accompanied by either the word ‘cause’ or ‘consequence’ and participants were told that their task was to retrieve that particular aspect of the memory (i.e., the cause or the consequence of the event) and to keep it in mind for 4000 ms whilst focusing on the cue. For the ‘no-think’ trials, participants attempted to keep the associated memory out of consciousness, and to block it from coming to mind whilst focusing on the cue word for 4000 ms.

Once the initial ATNT blocks had been completed, participants remained in the scanner and received additional instructions prior to undergoing two additional blocks of 72 trials (144 trials in total). The additional instructions informed participants that they would now see cue words which would be different from those presented in the first two blocks. They were told that these words would be presented in either red (‘no-think’) or blue (‘memory substitution’), with the blue cues accompanied by the words ‘cause’ or ‘consequence.’ Participants were told that, for the red cues, their task would be to do what they had done in the previous blocks and attempt to keep the associated memory out of consciousness whilst focusing on the word. For the blue cues, their task was to again keep the original learned memory from coming to mind but this time by thinking of the new memory (i.e., the memory which participants had learnt in the substitute learning phase), whilst paying attention to, and looking at the cue word the entire time. Participants were told that it was important to ensure they only used this method to ‘not think’ about the original memories for the blue and not the red cue words. The 144 additional trials comprised 12 presentations of six cue words in the second ‘no-think’ condition and six cue words in the ‘memory substitution’ condition^1^.

##### Final test phase (unscanned)

Once scanning had ended, participants were serially presented with all the cue words and told to disregard all previous instructions and to recall all of the associated memories in as much detail as possible. To reduce output interference, participants were told that, although they had learnt more than one memory for some of the cue words, it was very important that they recalled the original memory from the learning phase of the experiment first and then the second memory if they remembered it. Each cue word was presented for 30 s and participants were asked to press the spacebar as soon as the associated memory came to mind. Participants were then given 60 s in which to recall the cause and the consequence(s) of the event. All memories were recorded. As participants had learnt two memories for some cues, they were given up to 2 min to recall both sets of memories for these cues. Participants were again asked to rate all of the original memories in terms of their perceived valence, availability, imaginability, vividness, significance, and relatedness (see **Figure 1B**).

Finally, participants were given a post-experimental thought intrusions questionnaire in which they rated each of the no-think items in the direct suppression and memory substitution conditions. Participants were asked the degree to which they focused on the word as it appeared on the screen; how difficult they found it to ‘not think’ about the original memory associated with the word; how often the (original) associated memory came to mind; how often other thoughts (not including the alternative or substitute memory generated in session one) came to mind when the cue was presented and how often the second set (or substitute) memories came to mind when the cue was presented.

### Scoring and Behavioral Data Analysis

All the original memories were coded and scored using both strict and gist criteria (Noreen and MacLeod, 2013). For the strict criteria, a correct score was obtained if both descriptions concerning the cause and the consequence(s) for each memory were judged to correspond to the descriptions generated in session 1, with only minor variations being accepted as being correct. For the gist criteria, memories were scored as correct if the memory descriptions could be identified as referring to the same memories that were generated in the first session. A further independent coder was used to validate both the coding and the scoring by reading all the autobiographical memories from all participants (660 memories in total). For the coding, there was a high level of agreement between the two researchers (100% agreement for the gist criteria; 96% agreement for strict criteria overall; i.e., cause and consequence). Pearson correlations also revealed a strong correlation between the strict scoring of the memories by the two researchers [*r*(20) = 0.86, *p* < 0.01].

### fMRI Acquisition and Analysis

Scanning was performed on a 3T Siemens Trio whole-body MRI scanner (Siemens Medical Solutions, Erlangen, Germany) using a standard 12-channel receive-only whole-head coil. On-task functional data were acquired using a descending echo-planar pulse sequence (TR = 2000 ms, TE = 30 ms, 90° flip angle, 35 axial slices parallel to the AC–PC plane with 3.5 mm × 3.5 mm × 4 mm voxels, no inter-slice gap). Head motion was minimized using foam padding. High-resolution T1-, T2-, and FLAIR-weighted anatomical images were also acquired for visualization. All BOLD data were processed with SPM8. Slice acquisition timing correction was carried out by temporally resampling relative to the middle slice collected, followed by rigid body motion correction. Functional volumes were then spatially normalized to a canonical echo-planar template using 12-parameter affine and cosine basis transformations, and resampled to 3 mm isotropic voxels. Volumes were then spatially smoothed with a 6 mm Gaussian kernel.

#### Task-Evoked Univariate Analyses

Univariate analyses is the traditional method of rapid event-related fMRI analysis in which participants are treated as a random effect with volumes treated as a temporally correlated time series. Summary amplitudes were modeled by convolving a canonical hemodynamic response function with a series of delta functions marking the onset of each condition of interest. Trials were modeled as 4000 ms duration epochs from their respective onsets (the period for which they were asked to follow task instructions relating to the cue word). The best-fitting β parameter estimates of the canonical hemodynamic response function for each condition were used in pairwise contrasts and stored as a separate image for each participant. These images were tested against the null hypothesis of no difference between contrast conditions using one-tailed, repeated measures *t*-tests. Activations were considered significant and further scrutinized if they consisted of five or more contiguous voxels and exceeded an alpha threshold of 0.001 (typical threshold in memory research).

The two blocks consisting of ‘think’ and ‘no-think1’ trials and the two blocks consisting of ‘memory substitution’ and ‘no-think2’ trials were modeled in the same GLM but with separate beta parameter estimates for each condition according to block (i.e. two separate ‘no-think’ beta parameter estimates were made: ‘think-block no-think1,’ ‘memory substitution-block no-think2’). Summary amplitudes were also modeled separately for cause and consequence condition trials in both think and memory substitution blocks. Where ‘no-think’ conditions were contrasted with other active tasks, we restricted the no-think trials used to only those from the blocks corresponding to the active condition (e.g., the ‘think’ vs. ‘no-think1’ contrast used summary amplitudes for the no-think condition calculated using only no-think trials from blocks 1 and 2).

#### Region-of-Interest (ROI) Analyses

Region-of-interest (ROI) analyses were carried out to compare estimates of task-evoked response amplitude following initial exploration using univariate analyses. ROIs were defined as all voxels within a cluster from a univariate contrast or all voxels within a sphere of 5 mm diameter around a central coordinate, and were generated using the MARSBAR toolbox for SPM8 (Brett et al., 2002). We also used MARSBAR to extract each participant’s average response amplitude for each condition modeled in the GLM and then subjected these parameter estimates to standard inferential statistical analyses.

#### Psychophysiological Interaction (PPI) Analyses

We conducted a psychophysiological interaction (PPI) analysis to recover regions which selectively couple with an *a priori* defined seed region according to conditions defined in the GLM. This analysis was motivated by our hypothesis that increased activity in the right DLPFC region would couple with the hippocampus and exert a negative influence on hippocampal activation. For each participant, we conducted a single PPI using eigenvariate time series from the seed region centered on left posterior hippocampus. These time series were extracted using the VOI extraction function in SPM8 and then deconvolved with the canonical HRF (Gitelman et al., 2003). They were then multiplied by the psychological function (see below) and reconvolved with the HRF to obtain the PPI interaction term using the PPI function in SPM8. The PPI interaction terms were entered into each participant’s GLM as regressors of interest. The participant-level results were then taken to the random effects level in order to test the on-task network decoupling prediction outlined above.

Using a timecourse from the hippocampal seed as its physiological term, the psychological function considered think and no-think trials (e.g., set to -1 for think trials and +1 for no-think trials). The psychological function multipliers in the described states would yield regions whose timecourses were more correlated with the seed during no-think condition trials than during think condition trials. In these analyses, activations were considered significant and further scrutinized if they consisted of five or more contiguous voxels and if they exceeded an alpha threshold of 0.001.

## Results

### Characteristics of Autobiographical Memories

The mean ratings for the memories recalled regarding perceived availability, imaginability, significance, valence, vividness, and relatedness were each analyzed using a 2 (time; session 1 vs. session 2) × 2 (valence: positive vs. negative) × 5 (instruction; baseline vs. think vs. no-think1 vs. memory substitution vs. no-think2) mixed design analysis of variance (ANOVA). These analyses revealed that there was no effect of time on availability, *F*(2,41) = 2.28, *p* > 0.05 and relatedness, *F*(2,19) = 2.38, *p* > 0.05. There was also no effect of time on imaginability, significance, valence, and vividness; all tests *F* < 1.

In the remainder of this section, only significant effects are reported. Consistent with Baumeister et al. (2001) who suggest that negative memories have more impact than positive memories, our findings revealed participants’ perceived negative memories as being more significant to them than positive memories, *F*(1,20) = 12.67, *p* < 0.01, $\eta_{p}^{2}$ = 0.131 (*M* = 3.75, *SD* = 1.07 vs. *M* = 3.0, *SD* = 0.89). Furthermore, our analyses also revealed a significant time by valence effect, *F*(1,20) = 11.22, *p* < 0.01, $\eta_{p}^{2}$ = 0.118, with participants rating negative memories as more negative in session one than session two (*M* = 5.31, *SD* = 0.76 vs. *M* = 4.57, *SD* = 1.01); *t*(43) = 2.72, *p* < 0.01, *d* = 0.83. There were no significant differences, however, in the ratings of positive memories in sessions one and two (*M* = 2.09, *SD* = 0.94 vs. *M* = 2.62, *SD* = 1.07); *t*(43) = 2.0, *p* > 0.05, *d* = 0.53 (see **Table 1**).

**Table 1:** Showing mean ratings for characteristics of positive and negative memories in session 1 and session 2.

	Session 1 (mean *SD)*		Session 2 (mean *SD)*	
	
	Positive	Negative	Positive	Negative
Valence	2.09 (0.62)	5.31 (0.76)	2.61 (1.07)	4.57 (1.01)
Availability	2.51 (0.66)	2.76 (0.61)	2.74 (0.76)	3.0 (0.86)
Imaginability	2.56 (0.88)	2.71 (0.90)	2.64 (0.76)	2.92 (1.0)
Vividness	2.88 (0.93)	3.11 (0.85)	2.94 (0.73)	3.21 (0.94)
Significance	2.95 (0.94)	3.84 (1.06)	3.05 (0.85)	3.66 (1.10)
Relatedness	2.25 (0.53)	2.59 (0.66)	2.14 (0.64)	2.30 (0.65)

### Response Latency

In order to determine whether there were any differences in the mean latency for memories generated in session one, we conducted a 2 (valence: positive vs. negative) × 5 (instruction: baseline vs. think vs. no-think1 vs. memory substitution vs. no-think2) mixed design ANOVA. Consistent with previous findings obtained by Noreen and MacLeod (2013), there was no significant effect of instruction; all tests *F* < 1.

### Memory Accuracy

#### Memory Accuracy using the Strict Criteria

**FIGURE 2:**
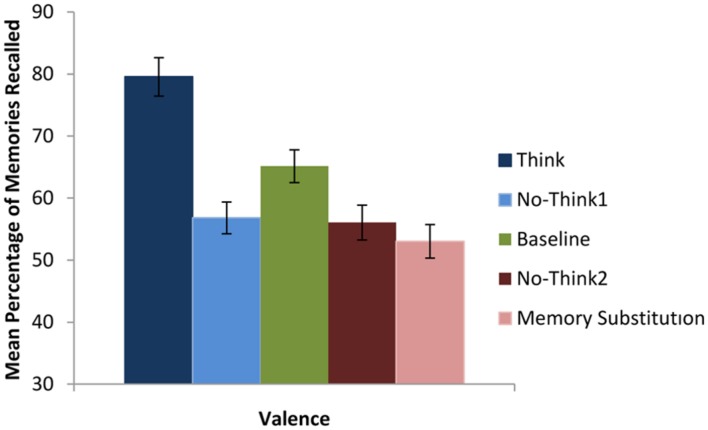
**Mean percentage of memories recalled as a function of instruction type**. Error bars represent ±1 standard errors of the mean (SEM).

In order to examine recall accuracy using the strict criteria, we conducted a 2 (valence: positive vs. negative) × 5 (instruction: baseline vs. think vs. no-think1 vs. memory substitution vs. no-think2) mixed design ANOVA. This analysis revealed a significant effect of instruction, *F*(5,16) = 15.79, *p* < 0.01, $\eta_{p}^{2}$ = 0.543 (see **Figure 2**). Subsequent pairwise analysis revealed that participants recalled significantly more memories in the think condition than in the never-presented baseline condition (*M* = 79.55, *SD* = 20.61 vs. *M* = 65.15, *SD* = 17.54); *t*(21) = 3.77, *p* < 0.01, *d* = 0.75. Participants also recalled significantly fewer memories in the no-think conditions than in the baseline condition (no-think1, *M* = 56.82, *SD* = 16.98 vs. *M* = 65.15, *SD* = 17.54; no-think2, *M* = 56.06, *SD* = 18.71 vs. *M* = 65.15, *SD* = 17.54); *t*(21) = 2.55, *p* < 0.02, *d* = 0.48, and *t*(21) = 2.61, *p* < 0.01, *d* = 0.50, respectively. Also fewer memories were recalled in the memory substitution condition than the baseline condition (*M* = 53.03, *SD* = 18.07 vs. *M* = 65.15, *SD* = 17.54); *t*(21) = 3.36, *p* < 0.01, *d* = 0.68. There was neither a significant effect of valence nor an instruction by valence interaction, all tests *F* < 1.

#### Memory Accuracy using the Gist Criteria

In order to examine recall accuracy using the gist criteria, we conducted a 2 (valence: positive vs. negative) × 5 (instruction: baseline vs. think vs. no-think1 vs. memory substitution vs. no-think2) mixed design ANOVA. This analysis revealed neither a significant effect of instruction, *F*(5,16) = 2.36, *p* > 0.05, valence, *F* < 1, *p* > 0.05, nor an instruction by valence interaction, *F*(5,16) = 1.47, *p* > 0.05. These findings are consistent with Noreen and MacLeod (2013) and suggest that although individuals may be successful at forgetting the details of memories, they do not forget the memory of the event itself (for a more detailed discussion on memory suppression using gist and strict criteria see Noreen and MacLeod, 2013).

### Thought Intrusions Questionnaire

Our analysis revealed that participants showed no significant differences in the degree to which they focused on the cue words in the direct suppression and the memory substitution conditions during the autobiographical think/no-think phase (*M* = 4.19, *SD* = 0.85 vs. *M* = 4.26, *SD* = 0.93); *t*(21) = 0.27, *p* > 0.05. We also found that there were no significant differences in how often the original associated memory came to mind in the direct suppression and the memory substitution conditions (*M* = 2.28, *SD* = 0.57 vs. *M* = 2.40, *SD* = 0.57); *t*(21) = 0.70, *p* > 0.05. We did, however, find that participants expressed greater difficulty in ‘not thinking’ about the original memory associated with the cue word in the direct suppression than the memory substitution condition (*M* = 2.90, *SD* = 0.98 vs. *M* = 2.24, *SD* = 0.87); *t*(21) = 2.39, *p* < 0.05, *d* = 0.71. Furthermore, participants also generated fewer alternative thoughts (not including the alternative memory generated in session 1) and fewer of the second set of memories in the direct suppression than the memory substitution condition; (*M* = 2.24, *SD* = 0.38 vs. *M* = 3.93, *SD* = 0.71); *t*(21) = 9.77, *p* > 0.01, *d* = 2.97; second set of memories (*M* = 1.61, *SD* = 0.46 vs. *M* = 4.48, *SD* = 0.44); *t*(21) = 21.45, *p* > 0.01, *d* = 6.38.

### Neural Correlates of Memory Retrieval and Suppression

The primary contrasts of interest were those showing differential activation across think and no-think1 conditions. Given the potential use of memory substitution as a strategy when following non-specific suppression instructions, we also examined activation differences across no-think2 and memory substitution conditions.

**FIGURE 3:**
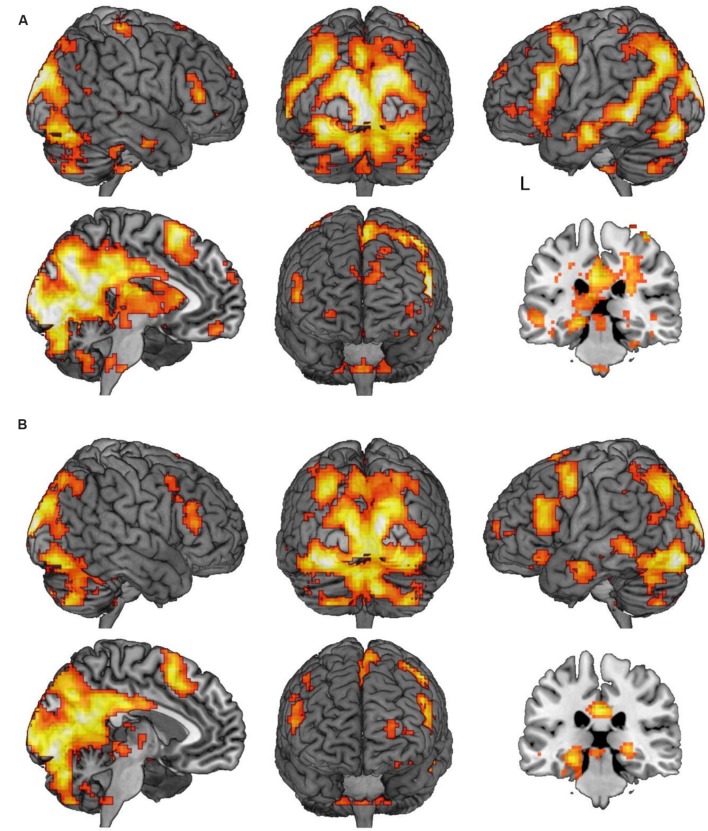
**Regions of activation in the think/memory substitution vs. no-think1 and 2 contrasts. (A)** Regions demonstrating significant activation in the think vs. no-think1 contrast. **(B)** Regions demonstrating significant activation in the memory substitution vs. no-think2 contrast. Images in both panels are thresholded at *p* < 0.001 with clusters comprising a minimum of five contiguous voxels. In both panels, the sagittal slice is marked with an L indicating the side of the left hemisphere and is rendered where *x* = -5. The coronal slice is rendered at *y* = -28.

We first sought to identify regions which were more active under instructions to ‘think’ than to ‘not think’ about memories associated with the cue words (think vs. no-think1). Unsurprisingly, under this contrast there was a great deal of activation (**Table 2** shows local maxima from the major cluster; **Table 3** shows a summary of the remaining clusters; **Figure 3A** renders the cortical activation on a canonical brain surface). A vast swathe of visual cortex activation extended along the midline and laterally into left parietal and temporal cortices. There was also a large region of activation extending laterally from the medial surface of left supplementary motor area to the pars triangularis of the inferior frontal gyrus. Suprathreshold subcortical structures included bilateral caudate and thalamus and left posterior parahippocampal formation and left posterior hippocampus. The opposite contrast (i.e., no-think1 vs. think) yielded a much more restricted activation map comprising one suprathreshold cluster in the right inferior parietal cortex (28 voxels in right BA 40, with peak activation at MNI coordinates [57, -55, 46]; not shown). Furthermore, we also conducted separate contrasts for no-think1 vs. think trials in blocks 1 and 2 which also revealed a similar pattern of activation^2^.

**Table 2:** Local maxima from the major cluster activated during the think vs. no-think1 contrast.

Region	Lat.	BA	*x*	*y*	*z*	Local maximum *Z* score
**Occipital**						
Lingual gyrus	L	18	-21	-79	-11	6.53
Calcarine sulcus	L	17	0	-88	-5	6.02
Superior occipital gyrus	L	17	-6	-103	10	5.96
	R	18	21	-94	28	5.47
Cingulum	R	26	9	-46	22	5.74
	L	23	-6	-49	22	5.71
Mid occipital gyrus	L	39	-39	-67	28	4.92
Calcarine sulcus	R	17	6	-58	16	4.90
	L	17	0	-97	1	5.55
**Frontal**						
IFG	L	45	-54	26	13	5.87
Precentral gyrus	L	6	-45	2	46	5.15
SMA	L	6	-3	17	61	5.13
MFG	L	8	-33	14	58	4.81
**Temporal**						
MTG	L	21	-60	-46	-2	6.07
Fusiform	R	18	24	-70	-11	5.91
Hippocampus	L	30	-18	-28	-8	4.58
ITG	L	20	-51	-19	-20	4.45
**Parietal**						
SMG	L	40	-36	-52	46	5.88
Precuneus	L	7	-6	-70	40	5.86
	R	23	21	-58	28	4.81
SPL	L	7	-27	-61	46	4.46
**Cerebellum**						
Posterior lobe	R	–	30	-73	-20	5.49
	R	–	24	-82	-17	4.93
Anterior lobe	L	–	-18	-55	-14	4.80

*Table summarizes local maxima within the major 13110 voxel cluster from the think vs. no-think contrast. Lat., laterality; BA, approximate Brodmann’s area; x, y, and z, coordinates in MNI space. IFG, Inferior frontal gyrus; MFG, middle frontal gyrus; SPL, superior parietal lobule; SMG, supramarginal gyrus*.

**Table 3:** Minor clusters activated during the think vs. no-think1 contrast.

Region	Lat.	BA	*x*	*y*	*z*	Vox.	Cluster *Z* score
**Frontal**							
OFC	R	47	30	50	-5	17	3.94
	L	11	-6	50	-17	23	3.76
	R	11	18	32	-14	6	3.34
	L	47	-39	50	-5	13	3.30
	L	34	-24	32	-17	7	3.57
Operculum	R	48	45	-7	19	18	3.92
SFG	L	32	-12	53	28	44	3.89
Insula	L	48	-33	-22	7	14	3.88
SMA	R	32	12	11	49	5	3.19
**Temporal**							
ITG	R	20	51	-10	-26	91	4.21
MTG	R	21	48	-34	-2	55	3.77
**Parietal**							
Post-central sulcus	R	3	42	-31	67	33	4.47
	L	2	-39	-40	67	7	3.35
AG	R	39	39	-64	34	8	3.35
**Cerebellum**							
Anterior lobe	R	20	30	-31	-29	22	3.81

*Table summarizes the remaining clusters not detailed in table from the think vs. no-think contrast. Lat., laterality; BA, approximate Brodmann’s area; x, y, and z, coordinates in MNI space; Vox., number of voxels within the cluster. OFC, Orbitofrontal cortex; SFG, superior frontal gyrus; SMA, supplementary motor area; ITG, inferior temporal gyrus; MTG, medial temporal gyrus; AG, sngular gyrus*.

We next sought to interrogate the differences in activation across no-think2 and memory substitution conditions. The memory substitution vs. no-think2 contrast yielded an activation map very similar to that obtained in the think vs. no-think1 contrast (**Table 4** shows local maxima from the major cluster; **Table 5** shows a summary of the remaining clusters; **Figure 3B** renders the cortical activation on a canonical brain) whilst the opposite contrast yielded no suprathreshold activations. Within the memory substitution vs. no-think2 contrast, all of the major cortical structures activated in the think vs. no-think1 contrast were again activated in the memory substitution contrast (though to a lesser extent, as evident in the decreased cluster contiguity). There were, however, some differences in the patterns of subcortical activity – decreased thalamus and caudate activation alongside maintained bilateral posterior parahippocampal and posterior hippocampal activations (local maxima at [-24, -31, -14] in the left hemisphere, [24, -28, -2] in the right). The correspondence in patterns of activation suggests that bringing to mind a target memory recruits support from a largely overlapping brain network to that recruited by bringing to mind an alternative memory using the same cue. Given that the major component of these two tasks is the controlled retrieval and maintenance of an autobiographical memory, this overlap in neural activation is unsurprising.

**Table 4:** Local maxima from the major cluster activated during the memory substitution vs. no-think2 contrast.

Region	Lat.	BA	*x*	*y*	*z*	Local maximum *Z* score
**Occipital**						
Lingual gyrus	L	18	-27	-85	-14	5.71
	R	18	24	-85	-14	5.18
Precuneus	L	23	-6	-61	19	5.40
	R	29	9	-49	19	5.36
Cuneus	R	17	12	-97	10	5.69
Calcarine sulcus	L	17	-3	-85	-11	5.43
Superior occipital gyrus	L	17	-9	-103	10	5.26
	R	18	21	-91	31	4.90
Inferior occipital gyrus	L	19	-42	-82	-11	4.45
	R	19	42	-82	-14	4.63
Mid occipital gyrus	L	7	-33	-67	40	5.08
	R	19	36	-79	10	3.79
Cingulum	L	23	-6	-49	34	4.80
**Temporal**						
Fusiform	L	37	-42	-58	-17	5.15
	R	19	27	-76	-8	5.02
MTG	L	21	-57	-43	-5	4.64
Parahippocampus	L	30	-24	-31	-14	4.60
**Parietal**						
Precuneus	L	7	-9	-67	37	5.10
SMG	L	40	-36	-52	37	5.08
Cingulum	R	23	0	-31	34	4.92
SPL	R	7	27	-76	49	4.14
Precuneus	R	23	18	-61	31	3.82
**Cerebellum**						
Anterior lobe	R	19	30	-76	-17	5.46
Posterior lobe	L	18	-15	-82	-23	4.50

*Table summarizes local maxima within the major 9078 voxel cluster from the memory substitution vs. no-think contrast. Lat., laterality; BA, approximate Brodmann’s area; x, y, and z, coordinates in MNI space. MTG, Middle temporal gyrus; SMG, supramarginal gyrus; SPL, superior parietal lobule*.

**Table 5:** Minor clusters activated during the memory substitution vs. no-think2 contrast.

Region	Lat.	BA	*x*	*y*	*z*	Vox.	Cluster *Z* score
***Frontal***							


OFC	L	47	-39	29	-14	84	3.94


	L	11	-24	35	-17	10	3.65


SFG	L	10	-24	62	13	14	3.64


Operculum	R	44	57	17	37	6	3.53


MFG	L	46	-39	53	7	7	3.41


SMA	L	6	-3	14	58	332	4.94


Precentral gyrus	R	6	48	8	52	27	3.59


	L	6	-45	2	49	550	4.94


***Temporal***							


MTG	L	21	-57	-4	-17	71	4.23


Fusiform	L	20	-36	-16	-32	12	3.55


***Brainstem***	–	–	-0	-37	-50	65	3.61

*Table summarizes the remaining clusters not detailed in **Table 4** from the memory substitution vs. no-think contrast. Lat., laterality; BA, approximate Brodmann’s area; x, y, and z, coordinates in MNI space, Vox., number of voxels within the cluster. OFC, Orbitofrontal cortex; SFG, superior frontal gyrus; MFG, middle frontal gyrus; SMA, supplementary motor area; MTG, medial temporal gyrus*.

**FIGURE 4:**
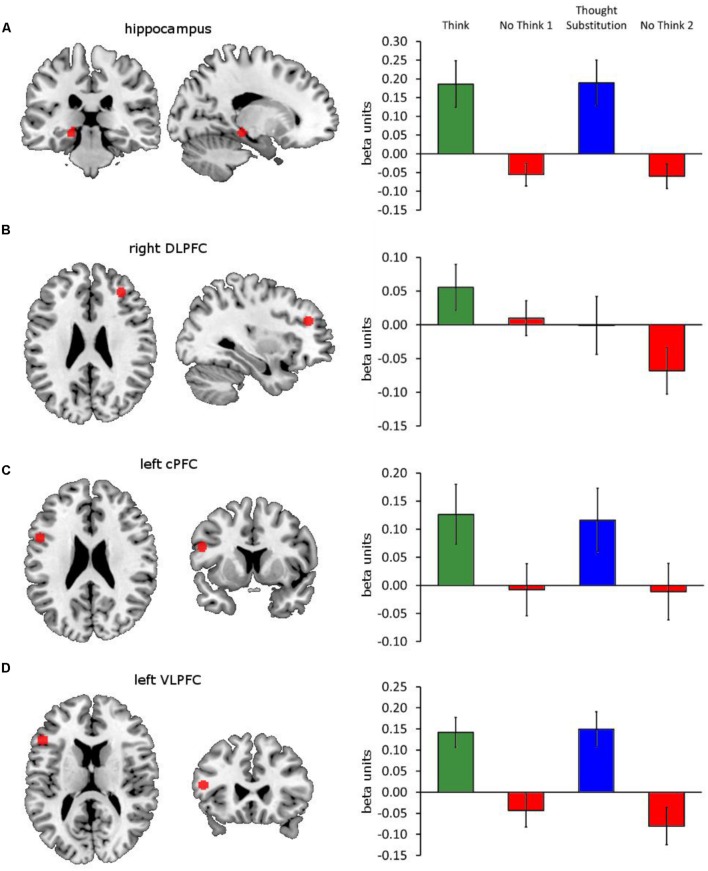
**Four ROIs and their beta amplitude responses**. Illustrations of the ROIs in red (left) and their beta amplitude responses to think, memory substitution, no-think1 and no-think2 (right) are shown for **(A)** left hippocampus, derived from a cluster within the think vs. threshold contrast thresholded at *p* < 0.0001, and 5 mm radius spheres centered on **(B)** right DLPFC [32, 38, 26], **(C)** left caudal PFC (cPFC) [-52, 9, 24] and **(D)** left VLPFC [-50, 25, 14] (BA 45). Error bars represent ±1 SEM.

We then examined the neural mechanisms by which the behavioral consequences of autobiographical memory suppression are enacted. Specifically, we wanted to establish whether the results of the subtraction contrasts above was brought about by activation in the think and memory substitution conditions, or deactivation in the no-think1 and 2 conditions, or via a combination of both. We also wanted to compare the activations obtained in this task to those reported in analogous tasks. To accomplish these checks and comparisons, we extracted the beta parameter and time course estimates from four ROIs (see **Figure 4**): the first was a 40 voxel left hippocampal cluster derived from the think vs. no-think1 threshold contrast thresholded at a conservative *p* < 0.0001; the next three were 5 mm radius spheres centered on right DLPFC [32, 38, 26] (BA 46), left caudal PFC (cPFC) [-52, 9, 24] (BA 44/6) and left VLPFC [-50, 25, 14] (BA 45). These four ROI were selected because they have previously been implicated in direct memory suppression and memory substitution (Benoit and Anderson, 2012). Furthermore, the DLPFC and the hippocampus regions have been robustly implicated in the suppression of a variety of different materials, including faces, words, and pictures, and therefore may also be involved in the suppression of autobiographical memories (Anderson et al., 2004; Depue et al., 2007; Benoit et al., 2014).

Repeated-measures comparisons of cause and consequence amplitudes within the think and memory substitution conditions, and no-think1 and no-think2 conditions (blocks 1–2 and blocks 3–4) yielded no significant differences, all *p*s > 0.10, so we collapsed across these pairs of conditions within these analyses (i.e., cause and consequence as well as no-think1 and no-think2). This yielded three sets of beta amplitudes – think (blocks 1–2), memory substitution (blocks 3–4), and no-think (blocks 1–4) – on which we conducted four repeated-measures one-way ANOVAs (one per ROI). **Figure 4** illustrates the ROIs and the beta amplitudes recovered from them under each of the conditions (note: no-think1 and no-think2 amplitudes are shown separately in the figure). Within the hippocampal ROI (**Figure 4A**), there was a significant difference in beta amplitudes according to condition, *F*(2,20) = 13.21, *p* < 0.001, $\eta_{p}^{2}$ = 0.386. Bonferroni-corrected pairwise comparisons revealed significant differences between the no-think condition (*M* = -0.06, *SD* = 0.12), and both think (*M* = 0.19, *SD* = 0.29), and memory substitution conditions (*M* = 0.19, *SD* = 0.29); both *p*s < 0.001. There was no significant difference in hippocampal response between the think and memory substitution conditions, *p* = 1.00. Given the finding in the literature that memory suppression is associated with hippocampal deactivation, we also conducted a single-sample *t*-test which revealed that the hippocampal response during the no-think condition was indeed significantly below baseline, *t*(21) = -2.31, *p* = 0.031, *d* = 0.637.

There were no significant differences in amplitude within the right DLPFC (**Figure 4B**), or the left cPFC (**Figure 4C**), *F*(2,42) = 1.82, *p* = 0.174, $\eta_{p}^{2}$ = 0.080, and *F* < 1, respectively. However, there was a significant difference across conditional amplitude responses in the left VLPFC (**Figure 4D**), *F*(2,20) = 15.26, *p* < 0.001, $\eta_{p}^{2}$ = 0.421. As with the left hippocampus, Bonferroni-corrected pairwise comparisons showed significant differences between the no-think condition (*M* = -0.06, *SD* = 0.14), and both think (*M* = 0.14, *SD* = 0.17); and memory substitution conditions (*M* = 0.15, *SD* = 0.20), both *p*s < 0.001, with no significant difference between the think and memory substitution conditions, *p* = 1.00. The VLPFC response during the no-think condition was not significantly below 0, *t*(21) = -2.01, *p* = 0.057, *d* = 0.430. These results are consistent with previous findings that demonstrate a significant deactivation in the hippocampus during the suppression of memories (Anderson et al., 2004; Benoit et al., 2014). More surprisingly, perhaps, the right DLPFC (a region associated with the exertion of cognitive control during this task; see Anderson et al., 2004; Depue et al., 2007; Benoit and Anderson, 2012; Levy and Anderson, 2012; Paz-Alonso et al., 2013) did not demonstrate elevated responses under no-think instructions, whilst the left cPFC and the VLPFC regions, which have both previously found to show elevated responses during memory substitution (Benoit and Anderson, 2012) also failed to demonstrate condition selectivity in their responding.

### Individual Differences in Forgetting

Given our finding that left hippocampal deactivation occurs during the no-think condition, we investigated whether the degree of hippocampal deactivation was associated with a behavioral outcome. Specifically, we hypothesized that individuals who were successful at forgetting would show greater hippocampal deactivation, operationalised as more negative beta amplitudes from the previously defined left hippocampal ROI, than individuals unsuccessful at forgetting. We classified participants as being either successful or unsuccessful at suppression based upon their recall performance on the final test. Successful suppressors were defined as individuals who demonstrated below-baseline forgetting on the final test, whereas unsuccessful suppressors were defined as individuals who had demonstrated equivalent or enhanced recall of memories associated with no-think cue words (in comparison to baseline). This resulted in 15 individuals who were successful at suppression and seven individuals who were unsuccessful at suppression. Pairwise analysis revealed that hippocampal response amplitudes were significantly lower for those individuals successful at forgetting (*M* = -0.12, *SD* = 0.09) than those individuals unsuccessful at forgetting (*M* = 0.05, *SD* = 1.0; *t*(21) = 3.68, *p* < 0.01, *d* = 1.68). There was no significant correlation, however, between hippocampal activation and the extent of forgetting, *r*(22) = 0.17, *p* > 0.05.

### Selective Coupling of Activation with Left Posterior Hippocampus

The findings presented so far suggest that deactivation in the left posterior hippocampal region may be associated with successful suppression of autobiographical memories. In spite of this, interrogation of *a priori* defined ROIs in the PFC failed to show a corresponding elevation in activation during no-think trials. As an alternative approach, we therefore searched for regions which might selectively engage with hippocampus during no-think trials whilst disengaging during think trials. To this end, we conducted a PPI analysis with the seed a 4 mm radius sphere centered on MNI coordinates [-21, -28, -8], a region of activation in left posterior hippocampus from the original think vs. no-think contrast which, on examination of amplitudes, was found to be driven by both think activation and no-think deactivation.

**FIGURE 5:**
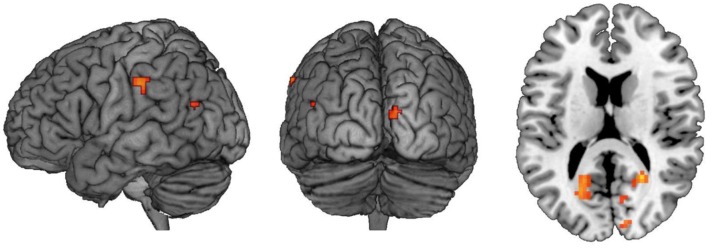
**Regions which show a no-think-couple/think-decouple relationship with left posterior hippocampus**. Results of a whole-brain PPI analysis using a 4 mm sphere centered on MNI coordinates [-21, -28, -8]. Regions highlighted couple with the seed region during the no-think condition and decouple during the think condition. Images are thresholded at *p* < 0.001 with clusters comprising a minimum of five contiguous voxels.

This analysis revealed a number of regions which selectively couple with the hippocampal seed during no-think trials and decouple during think trials (see **Figure 5**; **Table 6**). Besides those in occipital cortex and cerebellum, activations of note were present in left lateral parietal cortex, fusiform, and insular cortex. Notable absences of activation at this thresholding included the regions of cortex in the right hemisphere and lateral and medial prefrontal cortex. The exact relationship of the selectively coupled regions with hippocampus is unclear, although the rationale by which they have been recovered is indicative of greater engagement during suppress instructions (which are associated with deactivation in left posterior hippocampus) than during response instructions (associated with hippocampal activation).

**Table 6:** Regions which show a no-think-couple/think-decouple relationship with left posterior hippocampus.

Region	Lat.	BA	*x*	*y*	*z*	Vox.	Cluster *Z* score
**Occipital**							
Calcarine sulcus	R	17	24	55	16	26	4.34
	L	17/18	-21	67	19	56	3.92
	R	18	9	-73	19	5	3.28
Cuneus	R	18	12	-88	16	10	3.57
**Temporal**							
Fusiform	L	37	-33	-46	17	19	4.25
**Parietal**							
SMG	L	2	-63	25	37	18	3.79
AG	L	39	-45	64	22	5	3.33
**Cerebellum**							
Anterior lobe	L	–	-9	-40	50	8	3.72
**Frontal**							
Insula	L	48	-39	13	4	8	3.56

*Lat., laterality, BA, approximate Brodmann’s area; x, y, and z, coordinates in MNI space, Vox., number of voxels within the cluster. SMG, Supramarginal gyrus; AG, angular gyrus*.

## Discussion

The aim of our study was to identify the mechanisms associated with the suppression of meaningful and emotional autobiographical memories using the autobiographical think/no-think task (Noreen and MacLeod, 2013, 2014). Our behavioral findings indicated that, participants demonstrated comparable forgetting effects in the no-think (direct suppression) and the memory substitution conditions. These findings are consistent with a growing body of research which has demonstrated that, although individuals may use different strategies to prevent the unwanted memory from coming to mind, similar forgetting effects can be produced on tests which use the same cue to retrieve the unwanted memory (Bergström et al., 2009; Benoit and Anderson, 2012).

Despite demonstrating a forgetting effect in the no-think condition, however, we found no greater activation in the DLPFC during suppression. This finding is inconsistent with other studies which have found that direct suppression involves a highly demanding DLPFC-driven controlled process which overrides hippocampal retrieval (Anderson et al., 2004; Depue et al., 2007; Knoch and Fehr, 2007; Lieberman, 2007; Benoit et al., 2014; Gagnepain et al., 2014). Importantly, our finding is consistent with Hu et al. (2015) who examined the consequences of suppression instructions on autobiographical memories for a mock crime. Despite the fact that N200 is acknowledged to be sensitive to suppression and has been found to be predictive of successful forgetting (Bergström et al., 2009; Mecklinger et al., 2009), no frontal N200 activity was evident. Taken together, these findings may indicate that the suppression of autobiographical memories do not rely on the same pattern of fronto-hippocampal activation as the suppression of other less complex memories.

Interestingly, our findings also revealed a reduction in hippocampal activity during direct suppression. Consistent with reduced hippocampal activation being associated with increased suppression, we found that there was significantly lower hippocampal BOLD signal in those who were successful at suppressing unwanted memories compared to those that failed to show a forgetting effect. These particular findings provide partial support for the idea that there is reduced hippocampal activation during direct suppression (Anderson et al., 2004; Benoit and Anderson, 2012) and is consistent with the view that the hippocampus plays a key role in the retrieval (or not) of both personally meaningful and more abstract memoranda.

One possible reason for the absence of increased DLPFC activation in the no-think condition in the present study may relate to strategy use. In our study, all participants were given both direct suppression and memory substitution strategies to use during the ATNT phase. Thus, it is possible that participants may have used a combination of both strategies in each condition thereby contaminating our results with uncontrolled memory substitution in the no-think condition. It is important to mention here, however, that participants were only given the direct suppression strategy to use in the *first* two blocks of our study, thus mirroring the original task and representing as pure a measure of direct suppression as possible. The fact that we failed to find any significant differences between this ‘pure’ measure (as indexed by the first two ‘no-think’ blocks) and the potentially ‘contaminated’ measure of direct suppression (as indexed by the latter two ‘no-think’ blocks) – specifically, no increased activation in the DLPFC in the ‘pure’ no-think condition – would suggest that any cross-contamination of strategy use was minimal and therefore unlikely to have been a factor in explaining the contrast between the presents study and other related TNT studies.

A more plausible reason as to why our study did not find increased DLPFC activation for the ‘no-think’ trials in the direct suppression condition may be a function of having tapped into a different aspect of memory inhibition. The forgetting effects observed in our study are inherently different from those studies that have looked at suppression effects for other relatively simple stimuli such as words, pictures, or faces (Anderson et al., 2004; Depue et al., 2007; Benoit and Anderson, 2012; Benoit et al., 2014). Suppression effects in previous studies, for instance, have reflected impairment in the recollection of the unwanted target item on a subsequent memory test following suppression. In our study, in contrast, participants were unimpaired in their ability to recall the memory of the event itself but, rather, showed systematic forgetting effects for details associated with these memories. Thus, the form of forgetting for autobiographical memory may be much more subtle in its effects and may be related to the quality and specificity of the unwanted memory.

It is established that autobiographical memories are often emotional, vivid and rich with information and layers of varying complexity (Conway, 2001). Given the complex nature of these memories, it is possible that these memories comprise a number of components which may be differentially susceptible to the effects of suppression. Furthermore, given that we have found different observable effects of suppression for autobiographical memories – namely, forgetting effects for the details of these memories rather than the entire unwanted memory itself, it is possible that this form of suppression taxes control-related regions to a differing extent. Thus, the specific pattern of activation recruited in the frontal control related regions may vary according to task demands.

It is also possible that our failure to find DLPFC activation during suppression may be due to the fact that our trials contained a combination of both memories that were successfully suppressed and those that were unsuccessfully forgotten. Thus, it is possible that combining correct and incorrect suppression trials averaged out the effect that may have only been present for incorrect trials. It is important to mention, however, that we did look at individual differences in neural activation between successful suppressors (i.e., those that demonstrated below-baseline forgetting on the final test) and unsuccessful suppressors (i.e., those that showed equivalent or enhanced recall of memories relative to baseline recall). Overall, our findings revealed that although hippocampal response amplitudes were significantly lower for successful individuals than those individuals unsuccessful at forgetting, there was no difference in response amplitudes in the DLPFC. Furthermore, there was also no significant correlation between DLPFC activation and the size of the forgetting effect observed.

One notable strength of our study was that we used a within-subjects design. This allowed us to make a direct comparison between brain activation for the same individuals during direct suppression and memory substitution. This is especially important given that previous research has found that there are individual differences in the extent to which people can effectively suppress unwanted memories, with some individuals showing larger forgetting effects than others (Levy and Anderson, 2002; Noreen and MacLeod, 2013, 2014). As participants generated two sets of memories for the cue words in the first session, however, this opens up the possibility that interference from the irrelevant memory set may have occurred during direct suppression trials. It is important to mention here that participants were asked in the post-experimental questionnaire as to how often the second set of memories came to mind. This revealed that participants reported having experienced very little recall of the second set of memories during direct suppression (mean rating was 1.61 with 1 = never coming to mind and 5 = always coming to mind).

Our study also found that suppression in the no-think condition was associated with increased activation in the inferior parietal cortex. The inferior parietal region has already been implicated in memory retrieval (Wagner et al., 2005; Cabeza and St Jacques, 2007; Skinner and Fernandes, 2007; Ciaramelli et al., 2008; Vilberg and Rugg, 2008; O’Connor et al., 2010) and, more recently, research has explored how responses within the parietal region are modulated by task goals. Gimbel and Brewer (2014), for example, found that the parietal cortex (BA 40) showed increased activity (from baseline) during recall and direct suppression, thereby suggesting that the parietal lobe is highly sensitive to retrieval success, but only under conditions where retrieval has been attempted, with direct suppression abolishing any retrieval success.

Increased activity in the inferior parietal region has also been associated with action stopping (Aron et al., 2007; Swick et al., 2011) with inferior parietal regions appearing to mediate performance on tasks which require the inhibition of a motor response (Rubia et al., 2001). The fact that the parietal region has been implicated in both memory suppression and motor suppression is consistent with an inhibitory framework which suggests that the suppression of items from memory and motor stopping engage a common neural architecture, and that the apparent overlap between memory and motor systems means that one could expect to find generalized inhibitory control deficits in the population (Anderson, 2005; Levy and Anderson, 2008; Anderson and Weaver, 2009; Anderson and Huddleston, 2011). Clearly, further research is needed to fully understand the role of the inferior parietal cortex in the direct suppression of autobiographical memories.

Our findings also revealed that there was no increased activation in the cPFC and the VLPFC during memory substitution in comparison to the no-think condition. These findings are inconsistent with previous findings by Benoit and Anderson (2012) who reported increased cPFC and mid VLPFC activity during memory substitution. One reason why these regions did not demonstrate increased activity in the memory substitution condition in our study may relate to the fact that participants were asked to learn the substitute memory (instead of thinking about the originally associated memory) prior to scanning, which may have diminished competition between the original and substitute memory. Alternatively, it is possible that the lack of activity in the VLPFC may relate to the associative nature of autobiographical memory. The VLPFC is involved in the resolution of competition among multiple retrieved representations in memory and enables selected representations to guide decision and action (Badre and Wagner, 2007; Wimber et al., 2008). Given that autobiographical memory consists of a plethora of constructed memories, each containing event-specific knowledge with many associative connections between features, it is possible that, even under the direct suppression condition, the presentation of the cue word may have provided associative links to other memories. Thus, it is possible that cue words may not have been sufficiently distinct to discriminate a memory from alternatives.

It is important to mention here that one potential limitation of our study is the lack of trials used in the ATNT phase of our experiment. We had 288 critical trials with 6 × 12 trials per condition, whilst previous research has used in the order of 10 ∼ 15 × 12 trials per condition (Depue et al., 2007; Benoit and Anderson, 2012). Thus, it is possible that the apparent lack of an effect in the PFC could have been a function of low power. Whilst we accept this is a possibility, it remains unclear as to why our behavioral results have shown a successful forgetting effect whilst our neuroimaging results failed to find successful PFC activation. If suppression is indeed a result of a direct suppression mechanism, with DLPFC disengaging retrieval processes supported by the hippocampus then we could have expected DLPFC activation given the observed behavioral forgetting effect.

Another limitation to the current study is the fact that participants were asked in the first session to initially generate two memories for each cue word. Thus, it is possible that participants may not have engaged in direct suppression but may have used a memory substitution strategy instead and alternative memories that had been generated to prevent the target memory from coming to mind. Again, whilst we cannot rule out this possibility, it is important to note that we used real-life autobiographical memories which individuals had personally experienced. Autobiographical memory is acknowledged to be highly associative in nature, with generic cues often triggering multiple associated memories. Thus, while our study deviates from other suppression studies in this respect, the present study arguably provides a more realistic depiction of how suppression may actually operate in real-life.

The fact that we used ecologically valid memories of events that had occurred in individuals’ lives and used potent reminders to elicit these memories may ultimately help us to understand how memory control works in daily life, and thereby inform our understanding of clinical conditions. For instance, it is possible that by successfully suppressing some of the details associated with an unpleasant memory, it may be possible to gradually weaken that memory which may, in turn, help to reduce some of the associated painful emotions. This suppression process may also enable individuals to remember the event itself, which may have some associated adaptive value while promoting the loss of some of the more painful or distressing details associated with the trauma.

## Conclusion

Our findings revealed that, while participants demonstrated a forgetting effect in the no-think condition, they did not demonstrate greater activation in the DLPFC during direct suppression. Our findings, however, showed reduced hippocampal activation for those individuals who were successful at suppression, in comparison to those individuals who failed to demonstrate a successful forgetting effect. This finding suggests that direct suppression on hippocampal activity can disrupt a memory by impairing the quality and the specificity of it, even when the event itself remains accessible in memory. Our findings also revealed that direct suppression was associated with greater activation in the right inferior parietal region. This region has recently been implicated in direct suppression and motor stopping, providing support for the notion that memory stopping and motor stopping may engage in a common neural architecture. Finally, despite finding a forgetting effect in the memory substitution condition, we failed to find increased activation in the cPFC and VLPFC regions. It is clear from our findings that the suppression of autobiographical memory may require a much more nuanced approach if we are to fully understand how we keep particular autobiographical memories from coming to mind.

## Author Contributions

Conceived and designed the experiments: SN, AO, MM. Performed the experiments: SN. Analyzed the data: SN, AO. Wrote the paper: SN, AO, Critical revisions: SN, AO, MM.

## Conflict of Interest Statement

The authors declare that the research was conducted in the absence of any commercial or financial relationships that could be construed as a potential conflict of interest.

## Footnotes

1 It is important to mention here that the memory substitution and no-think trial blocks were always presented *after* the no-think and think trial blocks. This was to limit participant’s use of a substitution strategy during direct suppression so as to ensure as ‘pure’ a measure of direct suppression was obtained to ensure comparability with other TNT studies (Anderson and Green, 2001; Anderson et al., 2004).

2 As we only has 288 critical trials with 6 × 12 trials per condition and previous research has used trials in the order of 10 ∼ 15 × 12 trials per condition (Depue et al., 2007; Benoit and Anderson, 2012) it was not possible to look at the imaging data in terms of memory accuracy to determine whether the neural correlates of suppression differed depending on suppression success.
